# Photoelectric Dye, NK-5962, as a Potential Drug for Preventing Retinal Neurons from Apoptosis: Pharmacokinetic Studies Based on Review of the Evidence

**DOI:** 10.3390/life11060591

**Published:** 2021-06-21

**Authors:** Toshihiko Matsuo, Shihui Liu, Tetsuya Uchida, Satomi Onoue, Shinsaku Nakagawa, Mayumi Ishii, Kayoko Kanamitsu

**Affiliations:** 1 Department of Ophthalmology, Okayama University Hospital, Okayama City 700-8558, Japan; 2 Okayama University Graduate School of Interdisciplinary Science and Engineering in Health Systems, Okayama City 700-8558, Japan; shihuiliu@okayama-u.ac.jp; 3 Polymer Materials Science, Okayama University Graduate School of Natural Science and Technology, Okayama City 700-8530, Japan; tuchida@cc.okayama-u.ac.jp; 4 Laboratory of Biopharmacy, School of Pharmaceutical Sciences, University of Shizuoka, Shizuoka 422-8526, Japan; onoue@u-shizuoka-ken.ac.jp; 5 Laboratory of Biopharmaceutics, Graduate School of Pharmaceutical Sciences, Osaka University, Osaka 565-0871, Japan; nakagawa@phs.osaka-u.ac.jp; 6 Drug Discovery Initiative, The University of Tokyo, Tokyo 113-0033, Japan; mayumi-ishii@mol.f.u-tokyo.ac.jp (M.I.); kanamitsu@mol.f.u-tokyo.ac.jp (K.K.)

**Keywords:** NK-5962, photoelectric dye, apoptosis, retinal neuron, neuroprotection, pharmacokinetics, ADME, phototoxic/photosensitive assay, reactive oxygen species assay, photosafety

## Abstract

NK-5962 is a key component of photoelectric dye-based retinal prosthesis (OUReP). In testing the safety and efficacy, NK-5962 was safe in all tests for the biological evaluation of medical devices (ISO 10993) and effective in preventing retinal cells from death even under dark conditions. The long-term implantation of the photoelectric dye-coupled polyethylene film in the subretinal space of hereditary retinal dystrophic (RCS) rats prevented neurons from apoptosis in the adjacent retinal tissue. The intravitreous injection of NK-5962 in the eyes of RCS rats, indeed, reduced the number of apoptotic cells in the retinal outer nuclear layer irrespective of light or dark conditions. In this study, we reviewed the in vitro and in vivo evidence of neuroprotective effect of NK-5962 and designed pharmacokinetic experiments. The in vitro IC_50_ of 1.7 μM, based on the protective effect on retinal cells in culture, could explain the in vivo EC_50_ of 3 μM that is calculated from concentrations of intravitreous injection to prevent retinal neurons from apoptosis. Pharmacokinetics of NK-5962 showed that intravenous administration, but not oral administration, led to the effective concentration in the eye of rats. NK-5962 would be a candidate drug for delaying the deterioration of retinal dystrophy, such as retinitis pigmentosa.

## 1. Introduction

Retinitis pigmentosa is a hereditary disease which shows progressive loss of retinal photoreceptor cells usually at first in the peripheral retina. Patients with retinitis pigmentosa experience the slowly progressive constriction of the visual field caused by peripheral retinal dystrophy and frequently end up with the loss of the vision by the macular involvement. Up to this point in time, there has been no drug clinically available to stop or slow down the deterioration of retinitis pigmentosa. A best candidate would be a drug with safety in terms of long-term administration, which could prevent retinal neurons including photoreceptor cells from apoptotic death. A photoelectric dye, NK-5962, would be a candidate drug for this purpose. In this article, we review our previous results on neuroprotection with NK-5962 and present new data on pharmacokinetics of NK-5962.

## 2. Photoelectric Dye NK-5962

**Figure 1:**
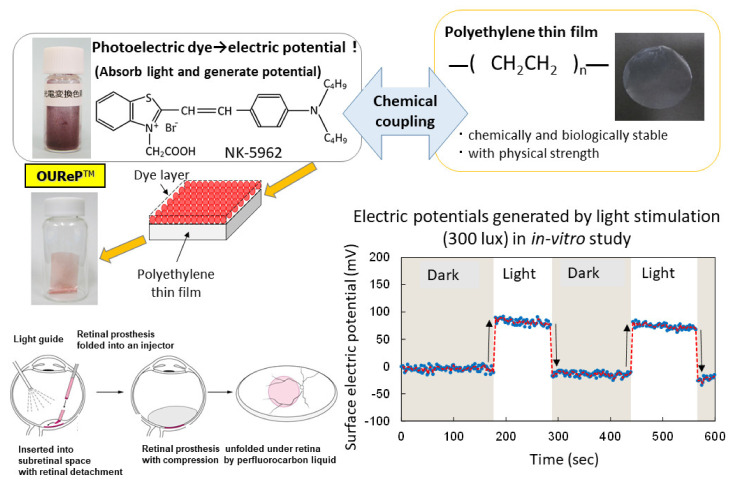
Overview of photoelectric dye-based retinal prosthesis (OUReP). Photoelectric dye NK-5962 molecules are chemically coupled to the polyethylene film surface. Light-evoked electric potential changes are recorded by a Kelvin probe on the dye-coupled film surface. The dye-coupled film can be implanted by a disposable injector (OUReP Injector) into the subretinal space of intentional retinal detachment during vitreous surgery.

A photoelectric dye, 2-[2-[4-(dibutylamino)phenyl]ethenyl]-3-carboxymethylbenzothiazolium bromide (NK-5962, Hayashibara, Inc., Okayama, Japan), generates electric potential in response to light and is used as a key component of photoelectric dye-based retinal prosthesis designated as OUReP (Okayama University Retinal Prosthesis) (Figure 1) [1,2,3]. The process of producing OUReP consists in thin films made from polyethylene powder and exposed to fuming nitric acid to introduce carboxyl moieties on the film surface. Photoelectric dye molecules are coupled to carboxyl moieties of the polyethylene film surface via ethylenediamine, as described previously [4,5,6]. The fuming nitric acid-treated only polyethylene film and the photoelectric dye-coupled polyethylene films were designated as the plain films and the dye-coupled films, respectively. Films were manufactured in a quality management system at a clean-room facility in Okayama University Incubator. No toxicity of the dye-coupled film was proven in all tests for the biological evaluation of medical devices, which is based on International Standard ISO 10993 (data not shown). In addition, the photoelectric dye NK-5962 was not toxic in the cytotoxicity test, skin sensitization test, genetic toxicity tests (bacterial reverse mutation test, human chromosomal aberration test), eye irritation test, and acute and repeated-dose systemic toxicity tests.

The dye-coupled films were proven to show light-evoked electric potential changes on their surface in the recording by Kelvin probe system [7,8]. The dye-coupled films were implanted in the subretinal space of hereditary retinal dystrophic rats (Royal College of Surgeons (RCS) rats) and behavior tests as well as electroretinographic and visual evoked potential recordings proved the efficacy of the dye-coupled films compared with the plain films [9,10,11,12]. As for the efficacy in in vivo large animal experiments, the dye-coupled films were implanted by vitreous surgery in the subretinal space of monkey eyes with macular degeneration and the visual evoked potential recordings that showed the recovery of the amplitude, which had been attenuated by the macular degeneration, in the 6 months after implantation [13]. As for the efficacy in in vitro testing, dystrophic retinal tissues isolated from RCS rats [8] and hereditary retinal dystrophic rd1 mice [14] were proven to induce light-evoked action potential spikes in the recording by a multielectrode array system. The other group of researchers recorded light-evoked changes in membrane action potentials in neurons, which were cultured on the NK-5962-conjugated indium tin oxide substrate surface [15]. As for safety, the dye-coupled films were implanted by vitreous surgery in the subretinal space of canine [16] and rabbit eyes [17] to prove surgical safety. Furthermore, disposable injectors were designed to implant the dye-coupled films into the subretinal space of the eyes in a safe and efficient manner [18,19]. From the viewpoint of materials science, polyethylene film surface was designed to induce lesser glial responses in the subretinal implantation [20].

In testing the safety of the photoelectric dye NK-5962, we found that the photoelectric dye, in itself, likely protected retinal neurons from death by apoptosis in vitro, even under dark conditions [21]. Furthermore, we noted that the long-term implantation of the photoelectric dye-coupled polyethylene film in the subretinal space of RCS rats prevented retinal neurons from apoptosis in the adjacent retinal tissue [10]. The intravitreous injection of NK-5962 in the eyes of RCS rats, indeed, reduced the number of apoptotic cells in the retinal outer nuclear layer irrespective of light or dark conditions [22].

In the following sections, we firstly review the in vitro evidence available [21] in Section 3 and Section 4. In Section 5, the in vivo evidence regarding the neuroprotective effect of NK-5962 in our previous study [22] is examined. We then present the design of pharmacokinetic experiments for NK-5962 as a candidate drug towards the neuroprotection in Section 6, demonstrated the pharmacokinetic results regarding the in vitro ADME assays in Section 7, and the in vivo administration in Section 8. We finally show the novel standardized testing for reactive oxygen species assay in Section 9. The following sections are independent with one another in that they have separate Methods and Results in each section. The structure of the article would allow readers to evaluate how the results were obtained by the use of different methods in each experiment.

## 3. Cytotoxicity Assay Using Mixed Culture of Retinal Neurons and Glial Cells

### 3.1. Methods

Egg shells (Fukuda Breeders Co., Ltd., Okayama, Japan) were disinfected with 70% alcohol and 12-day-old chick embryos were removed from a small opening. The eyes at this embryonic stage were used because the outer segments of the retinal photoreceptor cells had not yet been developed. The eyes were enucleated and cut at the midperiphery of the globe and the anterior halves were removed together with the vitreous. The neurosensory retina at this embryonic stage could easily be peeled off from the eye cup. After peeling, retinal tissues were incubated in 0.25% trypsin, 1 mM ethylenediaminetetraacetic acid (EDTA) in Ca^2+^, and Mg^2+^-free Hanks’ balanced salt solution (HBSS) to disperse retinal cells. After centrifugation, the retinal cells were washed with Dulbecco’s modified Eagle’s medium (DMEM) and seeded at a concentration of 3.5 × 10^5^ cell/mL in poly-L-lysine-coated wells of a 96-well multidish containing DMEM supplemented with 10% fetal bovine serum (FBS), 100 mg/L streptomycin, and 100 mg/L ampicillin.

**Figure 2:**
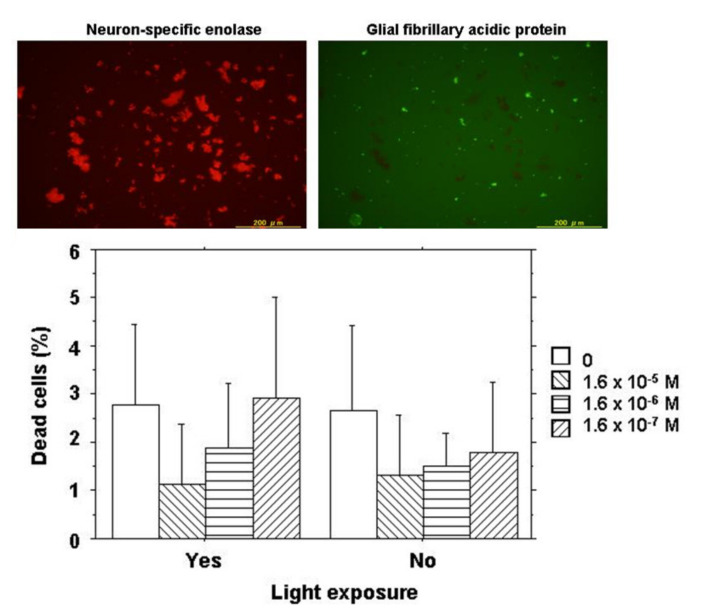
Cell viability assay for NK-5962 using chick retinal cells including neurons and glial cells. Top: Immunocytochemistry of retinal cells isolated from 12-day embryonic chick eyes and cultured for 2 days under light exposure for 9 h daily in the presence of NK-5962 at 1.6 × 10^−5^ M concentration. Neurons (red cells stained for neuron-specific enolase) in the left panel are the predominant population compared with a small number of glial cells (green cells stained for glial fibrillary acidic protein) in the right panel. Bottom: The percentage of dead retinal cells cultured for 2 days either under protection from light or under light with a fluorescent lamp at 230 lux for 9 h daily in the absence or the presence of NK-5962 at the concentrations of 1.6 × 10^−5^ M, 1.6 × 10^−6^ M, and 1.6 × 10^−7^ M. The percentage of dead cells is significantly smaller at a higher concentration of NK-5962 (*P* = 0.0183, two-factor ANOVA), while the percentage of dead cells is not significantly different between the dark condition and the light condition (*P* = 0.3102). T bars indicate standard deviation. Modified from reference [21].

In order to determine the types of cells, either neurons or glial cells derived from neurosensory retinal tissues, retinal cells following 2-day culture were stained for neuron-specific enolase and glial fibrillary acidic protein by immunocytochemistry. The majority of retinal cells at day 2 of the culture were neurons mixed with a small number of glial cells (Figure 2).

Retinal cells at a concentration of 3.5 × 10^5^ cell/mL in 220 μL of DMEM with 10% FBS were cultured together with 20 μL of varying concentrations of NK-5962 for 2 days either under protection from light or under light from a fluorescent lamp at 230 lux for 9 h daily. The final concentrations of NK-5962 in the solution were 1.6 × 10^−5^, 1.6 × 10^−6^, and 1.6 × 10^−7^ M. Cells were then incubated for 15 min with SYTO 10 Green fluorescent nucleic acid stain and DEAD Red nucleic acid stain (LIVE/DEAD Reduced Biohazard Viability/Cytotoxicity Kit: Molecular Probes; Thermo Fisher Scientific, Waltham, MA, USA) in HEPES-buffered saline. After being fixed with 4% glutaraldehyde in HEPES-buffered saline, live cells and dead cells were visualized with a 100 W mercury lamp as a light source under a fluorescence inverted microscope through a set of FITC and PI filters (Olympus IX71; Olympus Life Sciences, Tokyo, Japan). Images in the center of each well were captured with a CCD camera and stored in a computer. Three wells were assigned to each condition of incubation in one series of experiments and the entire procedure was repeated three times on different days to obtain nine results for each condition. Green live cells (stained with SYTO 10 Green) and orange dead cells (stained with both SYTO10 Green and DEAD RED) in the images for each well were counted and the percentage of dead cells to the total cells was analyzed statistically (Figure 2) [21].

### 3.2. Results

At day 2 of culture, live cells and dead cells were stained for cell viability and cytotoxicity analysis. The majority of retinal cells were alive with only a few dead cells under the dark condition or the light condition in the presence or the absence of NK-5962 (Figure 2). The percentage of dead cells was significantly smaller at higher concentrations of NK-5962 (*P* = 0.0183, two-factor analysis of variance (ANOVA), Table 1), while the percentage of dead cells was not significantly different between the dark condition and the light condition (*P* = 0.3102) [21]. 

## 4. Cytotoxicity Assay Using Retinal Pigment Epithelial Cells

### 4.1. Methods

**Figure 3:**
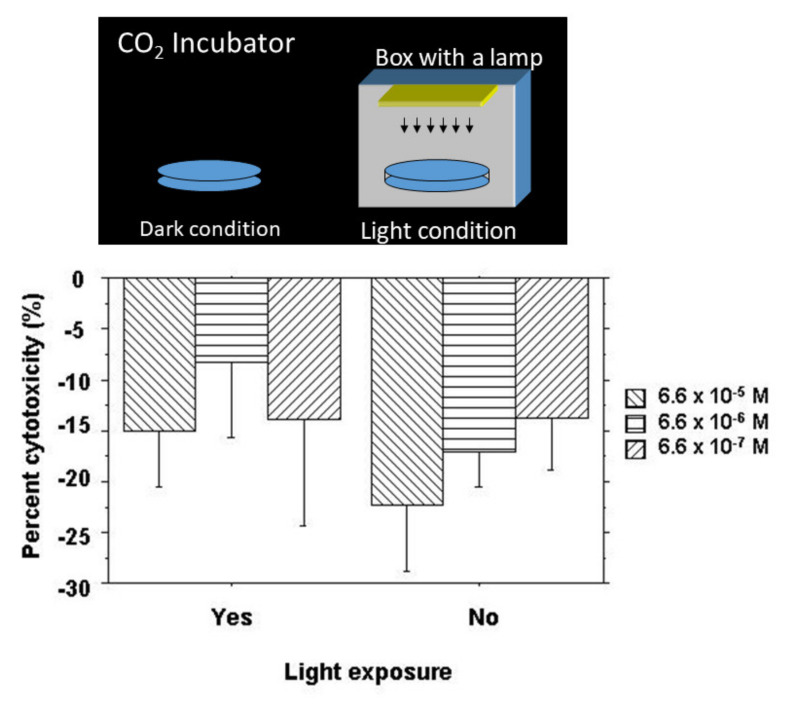
Cytotoxicity assay for NK-5962 using chick retinal pigment epithelial cells. Top: Standard dark condition in a CO_2_ incubator and light condition made by placing a culture dish inside a bottomless box with a lamp. Bottom: The percent cytotoxicity of retinal pigment epithelial cells incubated for 4 h with NK-5962 at the concentrations of 6.6 × 10^−5^ M, 6.6 x 10^−6^ M, and 6.6 × 10^−7^ M under the protection from light or under light with a fluorescent lamp at 320 lux for 4 h. The values in all groups are negative, indicating cytoprotective effects of NK-5962. Percent cytotoxicity values are significantly smaller under the dark condition (*P* = 0.0289, two-factor ANOVA) but do not reach statistical significance in comparison among three different concentrations of NK-5962 (*P* = 0.0849). T bars indicate standard deviation. Modified from reference [21].

The cytotoxic effect of NK-5962 was tested by a cytotoxicity assay using CytoTox 96 Non-Radioactive Cytotoxicity Assay (Promega, Madison, WI, USA) according to the manufacturer’s instructions. In order to isolate retinal pigment epithelial cells at the 12-day old chick embryonic stage, the retinal pigment epithelium in the eye cups, after the removal of the neurosensory retina, was incubated with 0.25% trypsin, 1 mM EDTA in Ca^2+^, and Mg^2+^-free Hanks’ balanced salt solution to peel off and disperse retinal pigment epithelial cells. Retinal pigment epithelial cells at a cell density of 2 × 10^4^ cells/100 μL of HEPES-buffered saline were incubated with 50 μL of varying concentrations of NK-5962 for 4 h at room temperature in wells of a V bottom 96-well plate either under protection from light or under light from a fluorescent lamp at 320 lux (Figure 3). The final concentrations of NK-5962 in the solution were 6.6 × 10^−5^, 6.6 × 10^−6^, and 6.6 × 10^−7^ M. After centrifugation of the plate at 250× *g* for 4 min, 50 μL supernatants were transferred to wells of a U-bottom 96-well assay plate. A substrate mix for lactate dehydrogenase (LD) enzymatic reaction in 50 μL of assay buffer (phosphate-buffered saline containing 1% bovine serum albumin) was added to each well and incubated for 30 min at room temperature under protection from light. The absorbance at 490 nm was read with an EIA microplate reader after the addition of stop solution (1 M acetic acid). The negative control was defined as spontaneous LD release (baseline) in the absence of NK-5962, while the positive control was defined as maximum LD release by the destruction of all cells in the presence of 10 μL of lysis solution (9% Triton X-100). All conditions were tested in four sets and a mean was obtained to calculate the percent cytotoxicity:

(measured value − baseline value)/maximum release) × 100

The procedure was repeated six times to obtain six sets of values for statistical analysis [21].

### 4.2. Results

Retinal pigment epithelial cells isolated from 12-day embryonic chick eyes were incubated with NK-5962 under the dark condition and the light condition for 4 h. The percent cytotoxicity value was negative in all groups (Figure 3), indicating protective effects by NK-5962. Percent cytotoxicity values were significantly smaller under the dark condition (*P* = 0.0289, two-factor ANOVA) but did not reach statistical significance in comparison among the three different concentrations of NK-5962 (*P* = 0.0849, Figure 3) [21].

## 5. Intravitreous Injection of NK-5962 in RCS Rats

### 5.1. Methods

**Figure 4:**
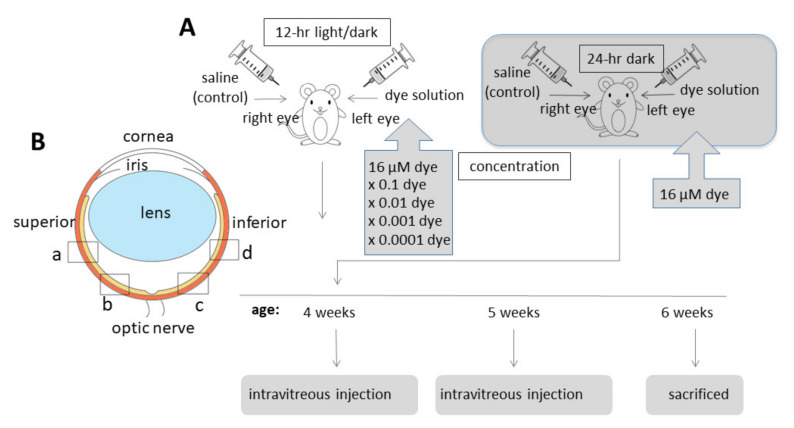
Intravitreous injection of NK-5962 in RCS rats’ eyes. (**A**) Time table for intravitreous injection of dye (NK-5962) solution in the left eye and control saline in the right eye of RCS rats twice at the age of 4 weeks and 5 weeks. (**B**) Four retinal sites defined for the analysis of apoptotic cell number. “a” and “b” begin at the straight distance of 373 μm and 160 μm, respectively, superior from the optic nerve head. “c” and “d” begin at the straight distance of 160 μm and 373 μm, respectively, inferior from the optic nerve head. Modified from reference [22].

NK-5962 powder was dissolved in distilled deionized water at a concentration of 8.2 μg/mL (16 μM). This original stock solution of NK-5962 (8.2 μg/mL) was diluted with saline (0.9% sodium chloride) to make a series of 10-fold dilutions from 8.2 × 10^−4^ to 8.2 μg/mL. Twenty male pink-eyed RCS rats (RCS/Jcl-rdy/rdy, p-, CLEA Japan, Inc., Tokyo, Japan) at the age of 4 weeks were assigned to 5 groups (n = 4) that received NK-5962 at 5 different concentrations. Intravitreous injection was performed twice at the age of 4 and 5 weeks (Figure 4A). This study was approved by the Animal Care and Use Committee at the Okayama University (Identifier OKU-2013441) and based on the Animal Welfare and Management Act in Japan.

RCS rats were anesthetized by intraperitoneal injection of ketamine (87 mg/kg body weight, Daiichi Sankyo, Tokyo, Japan) and xylasine (13 mg/kg, Bayer Japan, Osaka, Japan). Mydriasis was induced by 0.5% tropicamide and 0.5% phenylephrine instillation (Santen Pharmaceutical, Osaka, Japan) and corneal anesthesia was further obtained with 0.4% oxybuprokaine instillation (Santen Pharmaceutical, Osaka, Japan). Under a dissecting microscope, 3 μL of NK-5962 solution and saline (0.9% sodium chloride) was injected into the vitreous of the left eye and the right eye, respectively, with a 30-gauge needle attached to a Hamilton syringe (50 μL 1705 LT SYR; Hamilton Company, Reno, NV, USA). The needle was inserted almost at a perpendicular angle and at 1mm from the corneoscleral limbus to avoid the damage to the lens. Antibiotic (0.5% moxifloxacin, Novartis Pharma Japan, Tokyo, Japan) eye drops were instilled for both eyes. Each rat was housed in a standard rat cage in the 12 h light and dark cycle at the Animal Center of Okayama University. In a separate experiment, 3 rats were placed and maintained in constant darkness after intravitreous injection of NK-5962 at a concentration of 8.2 μg/mL (16 μM).

Apoptotic cells were detected by terminal deoxynucleotidyl transferase-mediated fluorescein-conjugated dUTP nick-end-labeling (TUNEL) assay, according to the manufacturer’s instructions (In Situ Cell Death Detection Kit, Roche Diagnostics, Mannheim, Germany). The images at the constant exposure time were captured by an Olympus FSX100 microscope that was equipped with the fluorescent filter cube U-MWIBA3. Five photographs in each of 5 retinal vertical sections per eye were taken and an entire frame (220 × 165 μm) of each photograph with ×40 NA0.95 objective lens was used for measurements. The number of TUNEL-positive cells in the outer nuclear layer at each retinal site (a, b, c and d, Figure 4B) was counted in each frame of photographs. The number of TUNEL-positive cells per 1,000 μm^2^ of the outer nuclear layer was calculated for comparison by two-factor ANOVA. TUNEL-positive cell numbers were compared among the left eyes with NK-5962 injection at different concentrations and the right eyes with control saline injection [22].

### 5.2. Results

**Figure 5:**
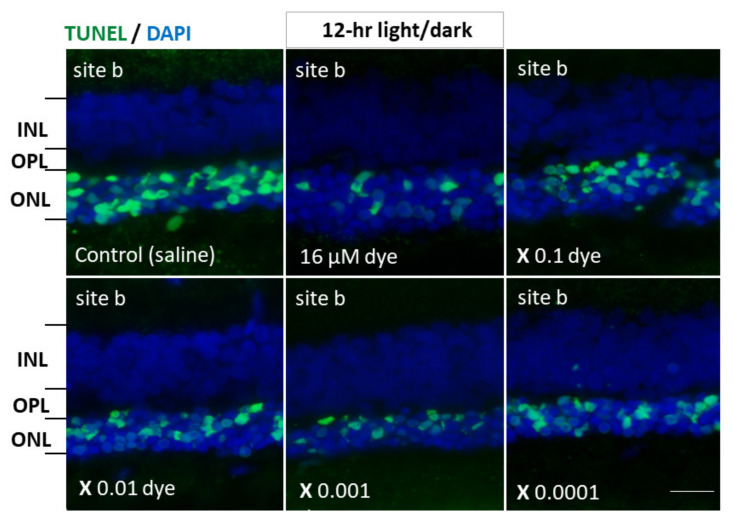
Apoptotic cells in retinal outer nuclear layer of RCS rats’ eyes with intravitreous injection of NK-5962 at 12 h light/dark condition. TUNEL staining (green) of retinal sections (site b) of the eyes with intravitreous injections of 3 μL saline in the right eye or series of dilutions of the dye (NK-5962) stock solution in the left eye under 12 h light/dark cycle. Eyes were enucleated two weeks after the first injection at the age of 4 weeks. The nuclei were counterstained with DAPI (blue). The number of TUNEL-positive cells in the outer nuclear layer (ONL) was less in the dye-injected eyes than in the saline-injected eyes. INL, inner nuclear layer; OPL, outer plexiform layer; ONL, outer nuclear layer. Scale bar = 10 μm. Modified from reference [22].

**Figure 6:**
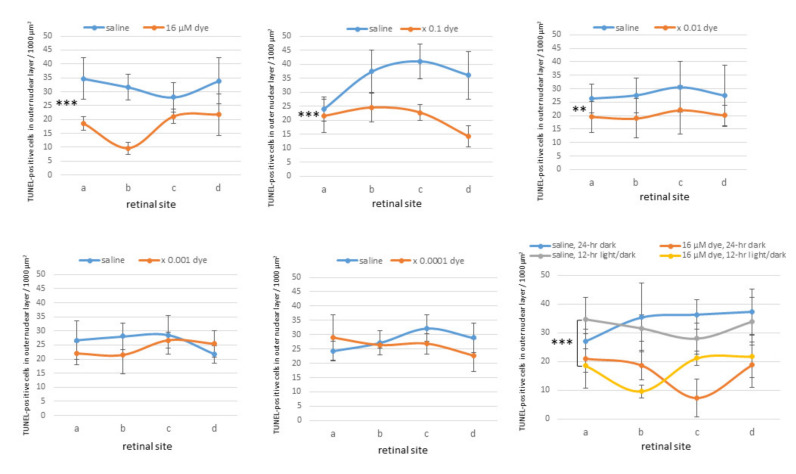
Quantitative analysis of apoptotic cells in the retinal outer nuclear layer of each group of rats. TUNEL-positive cell counts per 1000 μm^2^ in the outer nuclear layer of four different retinal sites (a, b, c, and d) of the left eye with dye (NK-5962) injection at five concentrations, compared to the right eye with saline injection under 12 h light/dark cycle. TUNEL-positive cell counts are significantly different among different dye (NK-5962) concentrations (*P* = 0.0001), but not significantly different among four different retinal sites (*P* = 0.144, two-factor analysis of variance, ANOVA). TUNEL-positive cell counts in dye (NK-5962)-injected eyes were significantly less than in saline-injected eyes at the concentration of 16 μM (*** *P* = 0.0001), 16 μM × 0.1 (*** *P* = 0.0001), and 16 μM × 0.01 (** *P* = 0.002). The bottom right panel shows TUNEL-positive cell counts in the eyes injected with dye (NK-5962 at 16 μM) versus saline under 12 h light/dark cycle and under 24 h constant dark condition. There is significant difference between dye (NK-5962)-injected eyes and saline-injected eyes under 24 h dark condition (*** *P* = 0.0001). T bars indicate standard deviation. Modified from reference [22].

Under 12 h light/dark cycle condition, in saline-injected eyes numerous TUNEL-positive cells were detectable in the outer nuclear layer where the nuclei of photoreceptor cells were located. In contrast, TUNEL-positive cells appeared to decrease in all four different sites (a, b, c and d) of retinal sections from NK-5962-injected eyes (Figure 5). TUNEL-positive cells in the outer nuclear layer were significantly different among different concentrations of NK-5962 (*P* = 0.0001), but not significantly different among four different retinal sites (*P* = 0.144, two-factor ANOVA). TUNEL-positive cells in the outer nuclear layer of the NK-5962-injected eyes were significantly less at three higher concentrations of NK-5962, ranging from 16 to 16 × 10^−2^ μM, compared to the saline-injected eyes (*P* = 0.0001, *P* = 0.0001, and *P* = 0.002, post hoc test, the least significant difference, Figure 6).

**Figure 7:**
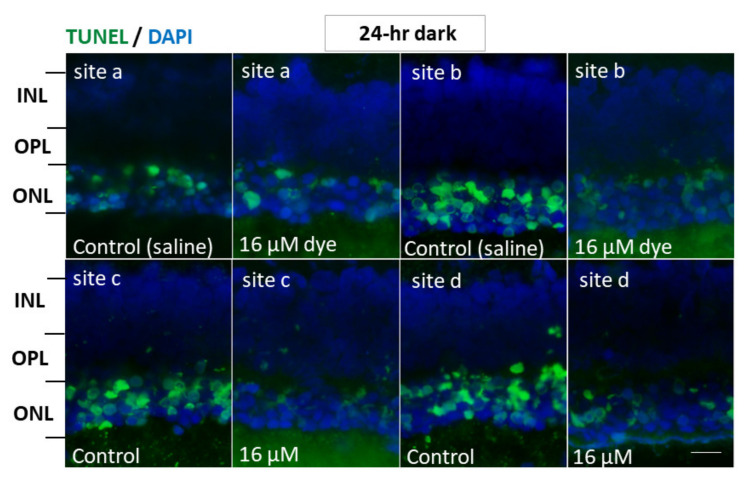
Apoptotic cells in retinal outer nuclear layer of RCS rats’ eyes with intravitreous injection of NK-5962 at 24 h dark condition. TUNEL staining of retinal sections at four different retinal sites (a, b, c, and d) in the left eye with dye (NK-5962 at 16 μM) injection, compared with the right eye with saline injection under 24 h constant dark condition. INL, inner nuclear layer; OPL, outer plexiform layer; ONL, outer nuclear layer. Scale bar = 10 μm. Modified from reference [22].

In another series of experiments, RCS rats injected with saline and NK-5962 at a highest concentration (16 μM) were kept under constant dark conditions (Figure 7). The number of TUNEL positive cells in the outer nuclear layer was significantly different between NK-5962 injection and saline injection (*P* = 0.0001), but not significantly different among four different retinal sites (*P* = 0.982, two-factor ANOVA). As post hoc tests, the number of TUNEL-positive cells in the outer nuclear layer of the NK-5962-injected eyes was significantly less than in the saline-injected eyes (*P* = 0.0001, Figure 6), but no significant difference was noted in the number of TUNEL positive cells between the constant dark condition and the 12 h light/dark cycle (*P* = 0.411) [22]. 

## 6. Designing of Pharmacokinetic Studies

According to the results of in vitro testing using chick retinal cells (Table 1) [21], the percentage of dead cells in the exposure to light was 2.78% in the absence of NK-5962, 2.93% in the presence of 0.16 μM NK-5962, 1.89% in 1.6 μM, and 1.17% in 16 μM. The rate of the percentage of dead cells in each concentration of NK-5962 when compared with the absence of NK-5962 was 2.93/2.78 = 105% in 0.16 μM NK-5962, 1.89/2.78 = 68% in 1.6 μM, and 1.17/2.78 = 42% in 16 μM. The IC_50_ (50% inhibition concentration) is thus located between 1.6 μM and 16 μM and is calculated as 4.9 μM, based on values at two points. Since the experimental cell-culture system contained 10% fetal bovine serum (FBS), the ratio of non-binding form of NK-5962 is 0.34 (Table 2). The IC_50_ based on free form of NK-5962 is thus calculated as 1.7 μM.

According to the results of intravitreous injection of NK-5962 in the eyes of RCS rats [22], the number of TUNEL-positive cells in the outer nuclear layer of the four retinal sites (a + b + c + d, Figure 4B) was used for calculation of EC_50_ (50% effective concentration). The percent ratio of the number of TUNEL-positive cells in 16 μM NK-5962 versus saline was 72/129 = 56% and the percent ratio of the number of TUNEL-positive cells in 1.6 μM NK-5962 versus saline was 85/139 = 61%. EC_50_ (50% effective concentration) would be around 16 μM. The intravitreous concentration following the injection of 16 μM NK-5962 in the amount of 3 μL was calculated as 16 μM × 3 μL / (3 + 13) μL = 3 μM, based on the estimation that the volume of the rat’ vitreous cavity would be 13 μL. Although the estimation of the IC_50_ and EC_50_, based on our previous studies [21,22], might be rough, the in vitro IC_50_ of 1.7 μM could, indeed, explain the in vivo EC_50_ of 3 μM.

## 7. In Vitro ADME Assay

### 7.1. Solubility Assay

The Japanese Pharmacopeia (JP) 1st fluid (pH 1.2) and JP 2nd fluid (pH 6.8) for dissolution testing were used for solubility measurements. The solution of a test compound was prepared by diluting 10 mM NK-5962 in 2 μL DMSO stock solution with 165 μL of JP 1st or 2nd fluid and then mixed at 37 °C for 4 h by rotation at 1000 rpm. After loading the mixed solution into 96-well MultiScreen Filter Plates (product number MSHVN4510, 0.45 μm hydrophilic PVDF membrane; Millipore, Bedford, MA, USA), filtration was performed by centrifugation. The filtrates were mixed with acetonitrile and analyzed by high-performance liquid chromatography-UV detection (HPLC-UV, 254 nm) or by liquid chromatography and tandem mass spectrometry (LC-MS/MS). Solubility was calculated by comparing the peak area of the filtrate mixture with that of a 100 μM standard solution.

### 7.2. PAMPA (Parallel Artificial Membrane Permeability Assay) 

In order to determine the passive membrane diffusion rates, the Corning Gentest Pre-coated PAMPA Plate System was used in the PAMPA test. The acceptor plate was prepared by adding 200 μL of 5% DMSO/100 mM phosphate buffer (pH7.4) to each well and then 300 μL of 0.2 μM test compound in 5% DMSO/100 mM phosphate buffer (pH6.4) was added to the donor wells. The acceptor plate was then placed on top of the donor plate and incubated at 37 °C without agitation for 4 h. At the end of the incubation, the plates were separated and the solutions from each well of both the acceptor plate and the donor plate were transferred to 96-well plates and mixed with acetonitrile. The final concentrations of compound in both the donor wells and acceptor wells, as well as the concentrations of the initial donor solutions, were analyzed by LC-MS/MS. The permeability of the compound was calculated according to the previous report [23]. The recovery of the test compound was more than 90%. The permeabilities of antipyrine (100 μM), metoprolol (500 μM), and sulfasarazine (500 μM) as reference compounds, with 100%, 95%, and 13% gastrointestinal absorption in human [23], were 11, 1.5 and 0.034 × 10^−6^ cm/s, respectively.

### 7.3. Hepatic Microsomal Stability Assay

The disappearance of the parent compound over time was measured by using the amount of drug at time zero as a reference. After 5 min of preincubation, 1 mM NADPH (final concentration, the same applies to the following) was added to a mixture containing 1 μM of the test compound, 0.2 mg/mL of rat or human liver microsomes (Sekisui XenoTech, LLC, Kansas City, KS), 1 mM EDTA, and 100 mM phosphate buffer (pH 7.4) and then incubated at 37 °C for 30 min by rotation at 60 rpm. An aliquot of 50 μL of the incubation mixture was sampled and added to 250 μL of chilled acetonitrile/internal standard (IS). After the centrifugation for 15 min at 3150× *g* at 4 °C, the supernatants were analyzed by LC-MS/MS. Hepatic microsomal stability (mL/min/kg, CLint) was calculated according to the previous report [24] and 44.8 or 48.8 mg microsomal protein/g liver and 40.0 or 25.7 g liver/kg body weight were used as scaling factors for the rat or human, respectively. 

### 7.4. Determination of the Unbound Fraction in Plasma and Medium

An equilibrium dialysis apparatus, Rapid Equilibrium Dialysis (RED) Device Single-Use Plate with Inserts, 8K MWCO (ThermoFisher Scientific, Waltham, MA, USA), was used to determine the unbound fraction for each compound in human plasma, rat plasma, and medium containing 10% fetal bovine serum (FBS). Plasma or medium was mixed with the test compound (1 μM) and 200 μL aliquots were loaded into the apparatus and dialyzed versus 350 μL of 100 mM phosphate buffer (pH 7.4) at 37 °C for 4 h by rotation at 1000 rpm. The unbound fraction was calculated as the ratio of receiver side (buffer) to donor side (plasma) concentrations.

### 7.5. Results of In Vitro ADME Assays

As shown in Table 2, the solubility of NK-5962 was low at pH 6.8. Therefore, the evaluation of membrane permeability was performed at 0.2 μM. The membrane permeability of NK-5962 by artificial membranes was lower than that of metoprolol, which has been reported to have 95% gastrointestinal absorption in humans [23]. The absorption of NK-5962 by oral administration was thus expected to be low and resulted in lower bioavailability. Furthermore, the permeability of the blood-ocular barrier was not expected to be large. When human and rat liver microsomes were used, NK-5962 showed moderate metabolic stability. This result was also considered to be partly responsible for the low bioavailability. NK-5962 was stable in the absence of the coenzyme NADPH, suggesting that it is mainly metabolized by cytochrome P450. The protein binding rate was not different between the humans and rats. Furthermore, based on the unbound fraction in 10% FBS, 0.34 times the concentration of the added compound was considered to contribute to the drug effect in vitro.

## 8. In Vivo Pharmacokinetics Assay

### 8.1. Intravenous and Oral Administration

Male Sprague Dawley (SD) and Wistar rats (5-week old) were obtained from Charles River Laboratories Japan (Yokohama, Japan) and Japan SLC, Inc. (Hamamatsu, Japan), respectively. All animals were maintained in air-conditioned quarters with room temperatures of 20 ± 2 °C, relative humidity of 50 ± 10%, and an alternating 12 h light/dark cycle. The rats were fed a certified diet MF (Oriental Yeast Co., Tokyo, Japan) and water ad libitum. The studies were conducted in accordance with the guidelines provided by the Animal Care and Use Committee of Osaka University (Identifier DouyakuR01-3-1).

A test compound (NK-5962) solution in 50% DMSO/water was injected intravenously (i.v.) to male SD rats. A test compound suspension in 0.5% methyl cellulose was administered orally (p.o.) to male Wistar rats. The dosage of the test compound was 1 mg/2 mL/kg (i.v.) and 10 or 100 mg/10 mL/kg (p.o.). Blood samples were collected from the jugular vein at 5 and 15 min and 1 h (i.v.) by using pre-heparinized syringe after the intravenous administration or from the caudal vein at 15 and 30 min, 1, 2 and 4 h (p.o.) by using heparinized capillary tube after the oral administration. At 30 min and 2 h (i.v.) or 8 h (p.o.), blood samples were collected from the abdominal aorta under isoflurane anesthesia and the eye balls and the whole brain were excised immediately.

Plasma was prepared by centrifugation of the blood samples. Plasma and tissues were stored at −80 °C. The tissues were homogenized with a four-fold volume of phosphate-buffered saline to obtain a 20% tissue homogenate. Plasma and 20% tissue homogenates were precipitated with 4–20 volumes of acetonitrile/IS and centrifuged at 15,000× *g* at 4 °C for 10 min. The supernatants were analyzed by liquid chromatography and tandem mass spectrometry (LC-MS/MS). Standard non-compartmental analysis was performed to determine the pharmacokinetic parameters: elimination half-life (t1/2) and area under the concentration time curve from time zero to infinity (AUC∞). The absolute bioavailability (BA) of the oral dose was calculated as

AUC∞ (p.o.)/AUC∞ (i.v.).

### 8.2. Topical Application as Eye Drops and Intravitreous Injection

The NK-5962 solutions at 16 μM concentration in phosphate-buffered saline and at 39.7 μM concentration in 0.5% methyl cellulose 400 solution (FUJIFILM Wako Chemicals, Osaka, Japan) were prepared as eye drops and topically applied to both eyes of two Wistar rats assigned for each concentration. The eyes were enucleated 30 min after topical application, washed twice in saline, weighed, and frozen for NK-5962 measurement. In parallel, 3 μL of the NK-5962 solution at 16 μM concentration in phosphate-buffered saline was injected into the vitreous of both eyes of 2 Wistar rats. The eyes in each rat were enucleated 5 min and 2 h after the intravitreous injection, washed twice in saline, weighed, and then frozen. Both eyes of one rat served as the negative control. This study was approved by the Animal Care and Use Committee at Okayama University (Identifier OKU-2016267, OKU-2019196).

### 8.3. LC-MS/MS Quantification Method for NK-5962

Liquid chromatography and tandem mass spectrometry instrument (LC-MS8060) equipped with Shimadzu Nexera series LC system (Shimadzu, Kyoto, Japan) were used. All compounds were analyzed in multi-reaction monitoring mode under electron spray ionization conditions. The analytical column used was a CAPCELLPAK C18 MGIII (3 μm × 2.0 mmID × 35 mm; OSAKA SODA, Osaka, Japan) at 50 °C. The gradient mobile phase consisted of 0.1% formic acid in water (mobile phase A) and 0.1% formic acid in acetonitrile (mobile phase B) at a total flow rate of 1 mL/minute. The initial mobile phase composition was 10% B, which was held constant for 0.2 min, increased in a linear fashion to 90% B over 1 min, then held constant for 0.8 min, and then finally brought back to the initial condition of 10% B over 0.01 min and re-equilibrated for 1 min. The transitions (precursor ion > product ion) of NK-5962 and IS (methyl testosterone) are 433.2 > 279.2 and 303.1 > 109.1 (positive), respectively.

### 8.4. Results of Pharmacokinetic Studies

**Figure 8:**
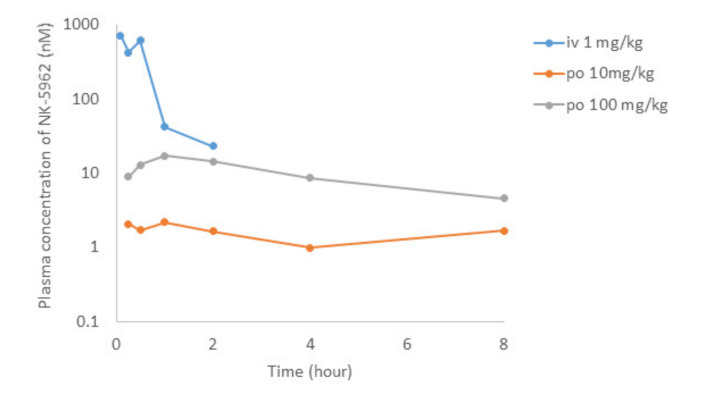
Pharmacokinetics of intravenous (i.v.) administration of NK-5962 1 mg/kg body weight and oral (p.o.) administration of NK-5962 10 mg and 100 mg/kg body weight in rats. NK-5962 concentrations in plasma are measured at designated time points. Graphs are created by excluding values which are suspected of contamination (Table 4).

After the intravenous administration of NK-5962 at 1 mg/kg body weight in rats, the plasma concentration of NK-5962 rapidly disappeared with the half-life of 0.35 h (Figure 8, Table 3). Although the eye ball concentration of NK-5962 was lower than the plasma concentration, the eye ball concentration at 30 min after the intravenous administration was 29.8 nM, which was about four times higher than the value of 7.25 nM at 5 min after intravitreous administration. Based on our previous study that 3 uL intravitreous dose of NK-5962 at the concentration of 16 μM had the efficacy, the 1 mg/kg intravenous administration could have the drug effect. On the other hand, the absolute bioavailability of NK-5962 in rats after the oral administration at 10 or 100 mg/kg was less than 1% (Figure 8, Table 4). The maximum plasma concentration after the oral administration of NK-5962 was 17 nM, excluding the 8 h values which were too high to be suspected of contamination and the eye ball concentration was expected to be lower. At this setting, using the eye ball/plasma concentration ratio of 0.072 for the 8 h value, the maximum eye ball concentration was expected to be as low as 1.3 nM. The eye ball concentration at 30 min after the topical application was 0.96 nM even at the higher dose of 39.7 μM. Based on these results, it is unlikely that the topical application up to 39.7 μM or the oral dose of 100 mg/kg might produce the drug effect.

## 9. Reactive Oxygen Species (ROS) Assay

### 9.1. Methods

The ROS assay was designed to detect both singlet oxygen and superoxide generated from photo-irradiated chemicals [25,26,27]. Briefly, singlet oxygen was measured in aqueous solution by spectrophotometrically monitoring the bleaching of p-nitrosodimethylaniline at 440 nm using imidazole as a selective acceptor of singlet oxygen. Samples containing the tested chemical (20 or 200 μM), p-nitrosodimethylaniline (50 μM), and imidazole (50 μM) in 20 mM sodium phosphate buffer (pH 7.4) were mixed in a tube. Each sample in 200 μL was transferred into a well of a plastic 96-well plate (clear, untreated, and flat-bottomed) and checked for precipitation under a microscope with an objective lens (×100) before light exposure. The plate was subjected to a measurement of absorbance at 440 nm using a microplate spectrophotometer. The plate was fixed in the quartz reaction container with a quartz cover and then irradiated with the simulated sunlight (ultraviolet A/B and visible light, 250 W/m^2^, Suntest CPS plus, Atlas Material Testing Technology, Mount Prospect, IL, USA) for 1 h. After agitation on a plate shaker, the UV absorbance at 440 nm was measured.

For the determination of superoxide, samples containing the test chemical (20 or 200 μM) and nitroblue tetrazolium (50 μM) in 20 mM sodium phosphate buffer were irradiated with the simulated sunlight for 1 h and the reduction in nitroblue tetrazolium was measured by the increase in absorbance at 560 nm in the same manner as the singlet oxygen determination. Experiments were performed in triplicate wells in three independent runs. As the final concentration, 200 μM test chemical solutions should be subjected to the ROS assay. However, when precipitation could be observed at 200 μM under the optical microscope, additional experiments should be performed under appropriate dilution (20, 50, or 100 μM). When precipitation was observed at 20 μM in the reaction mixture, further experiments were not required.

### 9.2. Results

NK-5962 and NK-4 (1-ethyl-4-[(1Z,3E,5E)-1-(1-ethylquinolin-1-ium-4-yl)-5-(1-ethylquinolin-4-ylidene)penta-1,3-dien-3-yl]quinolin-1-ium;iodide) [28,29,30,31,32] were tested in this study. Both NK-5962 and NK-4 showed a low level of reactive oxygen species generation (Table 5) and were concluded as less phototoxic from the viewpoint of drug candidates.

## 10. Conclusions and Future Perspectives

Based on our previous studies regarding the in vitro and in vivo efficacy of NK-5962 [21,22], we performed in vitro ADME studies and designed pharmacokinetic studies. As for the efficacy of NK-5962 in preventing retinal neuronal death, the in vitro concentration in the assay using chick retinal cells in culture [21] can explain the effective dose of intravitreous injection in the eyes of RCS rats [22]. The intravenous administration of NK-5962 led to the appropriate concentration in the eye ball which would show the efficacy whereas the oral administration did not result in the effective concentration in the eye ball. Topical application of NK-5962 as eye drops also did not result in the effective concentration in the eye ball. These facts suggest that NK-5962 in the blood could penetrate the blood–ocular barrier and that NK-5962 could be absorbed in a small quantity through the gut.

An ideal therapy for retinal dystrophy, such as retinitis pigmentosa, would be to stop the deterioration of the disease or at least to slow down the speed of the deterioration in a long-time span. At the moment, no drug is clinically available for this therapeutic purpose to prevent retinal neurons from death, probably by apoptosis. NK-5962 would be a candidate drug to have retinal neuroprotection and, hence, can delay the deteriorative changes in retinal dystrophy. In our previous in vitro and in vivo studies, the effect of NK-5962 to prevent retinal neurons from death by apoptosis did not depend on the presence of light [21,22]. NK-5962 is indeed a photoelectric dye, however, the efficacy to prevent retinal neurons from death by apoptosis would not be dependent on the effect of light. In this study, we showed additionally that NK-5962 did not have phototoxicity based on the novel standardized testing for reactive oxygen species assay [25,26,27]. Based on the lines of evidence, it can be assumed that the photoelectric property of NK-5962 would not be related with the effect of neuroprotection. We are in the ongoing process of transcriptome analysis by RNA-Seq in next generation sequencing to elucidate the molecular mechanism of neuroprotection induced by NK-5962.

Oral administration or topical application such as eye drops would be better routes for drugs and would be expected to have the efficacy in a long term to slow down deterioration in slowly-progressive chronic diseases, such as retinitis pigmentosa. However, the present pharmacokinetic studies showed poor penetrance of NK-5962 to the eye ball via oral administration and topical application with eye drops. The formula of eye drops with NK-5962 should be designed to enhance the intraocular penetrance to reach the effective concentration. High intraocular penetrance after the intravenous administration is a key factor in the design of a NK-5962 formula for oral administration.

Regarding the photoelectric dye-based retinal prosthesis (OUReP), an investigator-initiated clinical trial will be planned in patients with retinitis pigmentosa who have lost vision to light perception [33,34,35]. In parallel with the function of photoelectric dye-based retinal prosthesis, the neuroprotection by NK-5962 would be expected to play a role in maintaining the retinal tissue adjacent to the retinal prosthesis. In addition, NK-5962, as an adjunct therapeutic drug, would be administered to delay the deterioration of retinal dystrophy such as retinitis pigmentosa.

## Figures and Tables

**Table 1:** Live retinal cells and dead cells in each experiment under protection from light or under light for 9 h daily in the absence or the presence of NK-5962 at varying concentrations. Modified from reference [21].

		The Number of Dead Cells/The Number of Live Cells (The Percentage of Dead Cells in Total Cells)								
**NK-5962 Concentration**	**Light Exposure**	Well No. 1	Well No. 2	Well No. 3	Well No. 4	Well No. 5	Well No. 6	Well No. 7	Well No. 8	Well No. 9
0	Yes	13/242(5.0)	6/214(2.7)	9/257(3.3)	5/85(5.5)	2/138(1.4)	2/129(1.5)	3/133(2.2)	1/186(0.5)	8/272(2.9)
0	No	0/576(0)	17/457(3.5)	12/239(4.7)	4/166(2.3)	12/224(5.0)	1/247(0.4)	3/171(1.7)	10/253(3.8)	3/117(2.5)
1.6 × 10^−5^ M	Yes	0/254(0)	2/400(0.5)	1/397(0.3)	0/460(0)	4/207(1.9)	1/292(0.3)	5/153(3.2)	5/164(3.0)	2/152(1.3)
1.6 × 10^−5^ M	No	3/694(0.4)	0/287(0)	4/412(0.9)	9/584(1.5)	7/412(1.7)	4/533(0.7)	1/168(0.6)	3/141(2.1)	9/201(4.3)
1.6 × 10^−6^ M	Yes	1/185(0.5)	13/749(1.7)	1/478(0.2)	2/173(1.1)	5/239(2.0)	5/233(2.1)	6/148(3.9)	2/139(1.4)	6/140(4.1)
1.6 × 10^−6^ M	No	2/559(0.4)	7/552(1.3)	10/415(2.4)	2/240(0.8)	5/289(1.7)	2/150(1.3)	3/152(1.9)	2/138(1.4)	3/115(2.5)
1.6 × 10^−7^ M	Yes	11/231(4.5)	12/374(3.1)	0/247(0)	2/223(0.9)	3/123(2.4)	9/292(3.0)	10/167(5.6)	2/192(1.0)	10/158(5.9)
1.6 × 10^−7^ M	No	11/547(2.0)	2/652(0.3)	11/541(2.0)	3/241(1.2)	0/238(0)	7/294(2.3)	3/135(2.2)	2/153(1.3)	11/203(5.1)

**Table 2:** In vitro ADME evaluation of NK-5962.

Solubility (µM)		Membrane Permeability (×10^−6^ cm/s)	Microsome Metabolic Stability (mL/min/kg)		Protein-Binding (Non-Binding Ratio)		
JP1 (pH 1.2)	JP2 (pH 6.8)	PAMPA (pH 6.5)	Human	Rat	Human Plasma	Rat Plasma	10% Fetal Bovine Serum
79	0.2	0.31	68.3 (54.5 µL/min/mg)	272.2 (151.9 µL/min/mg)	0.028	0.022	0.34

**Table 3:** Eye ball and plasma concentrations of NK-5962 after single intravitreous injection, topical application with eye drops, or intravenous administration in rats.

Route of Administration	Concentration (Dose)	Rat ID-Eye	Time after Administration (Hour)	Eye Ball Concentration (nM)	Mean Eye Ball Concentration (nM)	Plasma Concentration (nM)	Eye Ball/ Plasma Ratio
Intravitreous	16 µM (3 µL)	1-left	0.083	9.23	7.25		
		1-right	0.083	5.27			
		6-left	2	1.19	1.49		
		6-right	2	1.78			
Topical (Eye drop)	16 µM (10 µL)	2-left	0.5	0.067	0.14		
		2-right	0.5	0.211			
		3-left (*dead)	0.5	0.222			
		3-right (*dead)	0.5	0.044			
Topical (Eye drop)	39.7 µM (10 µL)	4-left	0.5	1.55	0.96		
		4-right	0.5	0.510			
		5-left	0.5	1.19			
		5-right	0.5	0.592			
Intravenous	1 mg/kg body	1-iv	0.083			779	
		2-iv	0.083			648	
		1-iv	0.25			511	
		2-iv	0.25			342	
		1-iv	0.5	34.1	29.8	661	0.052
		2-iv	0.5	25.5		569	0.045
		3-iv	1			49.9	
		4-iv	1			34.6	
		3-iv	2	4.16	3.79	24.3	0.17
		4-iv	2	3.42		22.0	0.16

*dead indicates that this rat died from deep anesthesia before the extirpation of both eyes.

**Table 4:** Time profiles of NK-5962 concentrations in plasma and eye ball after single oral administration in rats.

Oral Dose (mg/kg Body)	Rat ID	Time after Administration (Hour)	Plasma Concentration (nM)	Eye Ball Concentration (nM)	Eye Ball/Plasma Ratio(Kp)
10	1-po	0.25	2.42		
	2-po	0.25	1.71		
	1-po	0.5	1.51		
	2-po	0.5	1.90		
	1-po	1	2.11		
	2-po	1	2.26		
	1-po	2	1.51		
	2-po	2	1.76		
	1-po	4	0.80		
	2-po	4	1.20		
	1-po	8	* 56.8	<0.1	Not calculated
	2-po	8	1.69	<0.1	<0.059
100	3-po	0.25	11.70		
	4-po	0.25	6.17		
	3-po	0.5	14.58		
	4-po	0.5	11.15		
	3-po	1	16.98		
	4-po	1	17.43		
	3-po	2	17.22		
	4-po	2	11.51		
	3-po	4	11.52		
	4-po	4	5.75		
	3-po	8	4.57	0.33	0.072
	4-po	8	* 44.4	0.40	Not calculated

* values are remeasured but deemed erroneous by contamination.

**Table 5:** Reactive oxygen species (ROS) assay for NK-5962 and NK-4.

Compound	Reactive Oxygen Species Generation	
	Singlet oxygen (ΔA440 × 1000)	Superoxide (ΔA560 × 1000)
	mean ± standard deviation (n = 3)	
Quinine 200 µM (Positive control)	602 ± 7	348 ± 12
Sulisobenzone 200 µM (Negative control)	Not detected	Not detected
NK-4 20 µM	26 ± 2	42 ± 6
NK-5962 20 µM	93 ± 14	Not detected

## Data Availability

Data in this study will be provided upon reasonable request to the corresponding author.
